# GPR139 and Dopamine D2 Receptor Co-express in the Same Cells of the Brain and May Functionally Interact

**DOI:** 10.3389/fnins.2019.00281

**Published:** 2019-03-26

**Authors:** Lien Wang, Grace Lee, Chester Kuei, Xiang Yao, Anthony Harrington, Pascal Bonaventure, Timothy W. Lovenberg, Changlu Liu

**Affiliations:** Janssen Research and Development, LLC, San Diego, CA, United States

**Keywords:** GPR139, dopamine D2 receptor, co-expression, *in situ* hybridization, *in vitro* interaction, calcium mobilization

## Abstract

GPR139, a G_q_-coupled receptor that is activated by the essential amino acids L-tryptophan and L-phenylalanine, is predominantly expressed in the brain and pituitary. The physiological function of GPR139 remains elusive despite the availability of pharmacological tool agonist compounds and knock-out mice. Whole tissue RNA sequencing data from human, mouse and rat tissues revealed that GPR139 and the dopamine D_2_ receptor (DRD2) exhibited some similarities in their distribution patterns in the brain and pituitary gland. To determine if there was true co-expression of these two receptors, we applied double *in situ* hybridization in mouse tissues using the RNAscope^®^ technique. GPR139 and DRD2 mRNA co-expressed in a majority of same cells within part of the dopaminergic mesolimbic pathways (ventral tegmental area and olfactory tubercle), the nigrostriatal pathway (compact part of substantia nigra and caudate putamen), and also the tuberoinfundibular pathway (arcuate hypothalamic nucleus and anterior lobe of pituitary). Both receptors mRNA also co-express in the same cells of the brain regions involved in responses to negative stimulus and stress, such as lateral habenula, lateral septum, interpeduncular nucleus, and medial raphe nuclei. GPR139 mRNA expression was detected in the dentate gyrus and the pyramidal cell layer of the hippocampus as well as the paraventricular hypothalamic nucleus. The functional interaction between GPR139 and DRD2 was studied *in vitro* using a calcium mobilization assay in cells co-transfected with both receptors from several species (human, rat, and mouse). The dopamine DRD2 agonist did not stimulate calcium response in cells expressing DRD2 alone consistent with the G_i_ signaling transduction pathway of this receptor. In cells co-transfected with DRD2 and GPR139 the DRD2 agonist was able to stimulate calcium response and its effect was blocked by either a DRD2 or a GPR139 antagonist supporting an *in vitro* interaction between GPR139 and DRD2. Taken together, these data showed that GPR139 and DRD2 are in position to functionally interact in native tissue.

## Introduction

GPR139 (aka GPRg1 or GPCR12) was originally cloned and characterized as a novel Gq-coupled orphan receptor (Liu et al., 2015). Several groups have reported surrogate ligands for GPR139, including TC-O 9311 [3,5-dimethoxybenzoic acid 2-[(1-naphthalenylamino)carbonyl]hydrazide] and JNJ-63533054 as potent and selective agonists (Hu et al., 2009; Shi et al., 2011; Dvorak et al., 2015; Wang et al., 2015). Based on known surrogate agonists, Isberg et al. (2014) disclosed a pharmacophore model and proposed that L-tryptophan (L-Trp) and L-phenylalanine (L-Phe) were the putative endogenous ligands for GPR139. Simultaneous to this, we provided additional and independent biological and pharmacological evidence to support that L-Trp and L-Phe activated GPR139 and supported their likelihood as endogenous ligands (Liu et al., 2015). Despite the availability of several selective agonists, the physiological function of GPR139 remains elusive. Its potential role in addiction (Kononoff et al., 2018), Parkinson’s disease (Bayer Andersen et al., 2016), locomotor activity (Liu et al., 2015), and schizophrenia (Atienza et al., 2018) has been suggested. L-Trp and L-Phe are the precursors for serotonin and dopamine biosynthesis, respectively, which are neurotransmitters implicated in mood regulation (Toker et al., 2010; Anjema et al., 2011; ten Hoedt et al., 2011; Jenkins et al., 2016). The amino acid sequence of GPR139 is highly conserved across species (Liu et al., 2015). Expression of GPR139 is almost exclusive to the central nervous system (CNS), except for expression observed in the pituitary (Matsuo et al., 2005; Susens et al., 2006; Liu et al., 2015). GPR139 mRNA is highly expressed in the lateral septum and medial habenula, and GPR139 immunoreactivity is also detected there (Liu et al., 2015). Relatively high expression of GPR139 mRNA is also found in the lateral caudate, zona incerta, and medial mammillary nucleus (Matsuo et al., 2005). As we compared the RNA sequence data between GPR139 and other known GPCRs in human, mouse, and rat tissues, we noticed that GPR139 and dopamine D_2_ receptor (DRD2) exhibited similar distribution patterns in the brain as well as in the pituitary gland across several species. Therefore, we investigated the possible co-expression of GPR139 and dopamine DRD2 mRNA in the same cells of brain as well as in pituitary by using RNAscope^®^ double *in situ* hybridization in mouse tissue sections. We further explored a potential *in vitro* interaction between GPR139 and DRD2 by performing co-transfection experiments.

## Materials and Methods

### Compounds

Quinpirole (Quinpirole hydrochloride) was purchased from Sigma-Aldrich (St. Louis, MO, United States). L-741,626 [3-[4-(4-Chlorophenyl)-4-hydroxypiperidin-l-yl]methyl-1H-indole] was purchased from Tocris Bioscience (Bristol, United Kingdom). JNJ-3792165 [1H-pyrazole-3-carboxamide, 1-[(2,6-dichlorophenyl)methyl]-5-methyl-*N*-(3-methylphenyl)]and JNJ-63533054 [(S)-3-bromo-5-chloro-N-[2-oxo-2-[(1-phenylethyl)amino]ethyl] benzamide] were synthesized at Janssen Research and Development, LLC (San Diego, CA, United States) as previously described (Dvorak et al., 2015).

### RNA Sequencing

Human RNA sequencing raw data of adult non-tumor tissue samples were obtained from the genotype-tissue expression (GTEx) project (GTExConsortium, 2015) version 6 release. Additional human tissue samples from individual donors were acquired from BioChian (Newark, CA, United States). Individual mouse samples (C57BL6, 10–12 weeks old, 5 mice for each tissue) were from Zyagen (San Diego, CA, United States). Rat pooled tissue samples (Sprague-Dawley, 10 weeks old, ≥4 rats for each tissue) were from Harlan Laboratories (Indianapolis, IN, United States). All samples were sequenced by BGI Americas (Cambridge, MA, United States) with the Illumina HiSeq (San Diego, CA, United States) platform using the same protocols applied in GTEx project. All RNA sequencing data were processed using Omicsoft tools (Cary, NC, United States). RNA sequencing reads were mapped onto human GRCh38, mouse GRCm38 and rat Rnor_5.0 genome assemblies. Ensemble gene models were applied to represent genes in each genome. Transcripts per kilobase million (TPM) was calculated to determine gene expression in each sample and to normalize across samples (Mortazavi et al., 2008). Samples with failing raw data and mapping quality control or having low consistency with other samples of the same tissue type, were removed from the final data calculations.

### Tissue Preparation

Mouse coronal brain sections were obtained from Abbomax (San Jose, CA, United States). Mouse sagittal pituitary bulb sections were obtained from Zyagen (Cat. No. MP-502, San Diego, CA, United States). Both tissues were collected from total 3 adult C57BL/6 mice with gender randomly selected. Tissues were freshly harvested, fixed in 10% neutral buffered formalin solution (NBF), processed for paraffin embedding, and sectioned onto SuperFrost Plus slides (Fisher Scientific, Waltham, MA, United States) at 5 μm thickness. To reduce autofluorescence, the sectioned tissue samples were irradiated with UV light (30 Watt, 253 to 400 nm discrete emission, Philips, Amsterdam, Netherlands) at room temperature for 2 h before the *in situ* hybridization procedure.

### RNAscope^®^ *in situ* Hybridization

RNA *in situ* hybridization experiments were performed using RNAscope^®^, an RNA *in situ* hybridization technique described previously (Wang et al., 2012). The RNAscope^®^ Fluorescent Multiplex Reagent Kit (Cat. No. 320850, Advanced Cell Diagnostics, Newark, CA, United States) was used according to the manufacturer’s instructions. Paired double-Z oligonucleotide probes were designed against target RNA using custom software to specifically detect the genes of interest. The following probes were used in this study: Mm-Gpr139 (Cat. 318051) and Mm-Drd2-C2 (Cat. 406501-C2). Probes were assigned to the following channel and fluorophore configuration: Mm-Gpr139-C1 (Cy3)/Mm-Drd2-C2 (Cy5). Formaldehyde fixed-paraffin embedded tissue section samples were sourced by ACD, and each sample was evaluated for RNA integrity with the positive control probes Mm-Ppib-C1 (Cat. 313911)/Mm-Polr2a-C2 (Cat. 312471-C2) and a 2-plex *dapB* negative control probe (Cat. 320751). Using the positive and negative control probes, optimization was performed to establish the following pretreatment conditions: 15 min target retrieval at 95–100°C and 30 min Protease Plus at 40°C. Staining was performed at ACD Newark by the Pharma Assay Services group. The nuclei of cells were counterstained with DAPI (4’,6-diamidino-2-phenylindole) nuclear stain before the slides were cover slipped. For brain slides, fluorescent images were acquired using a Zeiss LSM 700 confocal microscope. Confocal images were then processed and analyzed using Zen Lite 2.3 software (Blue Edition, Carl Zeiss Inc., Oberkocken, Germany). The pituitary slides were scanned and digitalized by using a Pannoramic SCAN II scanner (3DHISTECH Ltd, Budapest, Hungary) equipped with 40x objective lens, sCMOS color camera with 6.5um pixel size (pco.edge 4.2, PCO-Tech, Romulus, MI, United States) and a solid-state light source (Spectra X light engine, Lumencor, Beaverton, OR, United States). Resulting images were processed and analyzed with CaseViewer 2.1 software (3DHISTECH Ltd, Budapest, Hungary) and Zen Lite 2.3 software (Blue Edition, Carl Zeiss Inc., Oberkocken, Germany). GPR139 and DRD2 mRNA signals are illustrated with red and white color in the images, respectively.

### Molecular Cloning of GPR139 and Dopamine D2 Receptor

GPR139 from human, mouse and rat were cloned from respective brain cDNAs as previously described (Liu et al., 2015). DRD2 from human, mouse and rat were cloned from respective brain cDNAs. Specific primers (Forward primer: 5’- atg tca GAA TTC GCC ACC atg gat cca ctg aat ctg tcc tgg tat gat-3’; Reverse primer: 5’- tga cat GCG GCC GCt cag cag tga agg atc ttc agg aag gcc ttg cgg aa-3’) designed based on published human DRD2 cDNA coding region (Genbank Accession NO: M30625.1) were used for PCR amplification of the human brain cDNA (Clontech) using an Expanded High Fidelity PCR System (Roche). The resulting PCR product was cloned into pcDNA3.1 between EcoR1 and Not1 sites (Promega, Madison, WI, United States). The insert region was sequenced by Eton Biosciences (San Diego, CA, United States) to verify the identities. Similarly, mouse and rat DRD2 cDNA were PCR amplified using primers (mouse, Forward primer: 5’- atg tca GAA TTC GCC ACC atg gat cca ctg aac ctg tcc tgg tac-3’, Reverse primer: 5’- atg tca GCG GCC GCt cag cag tgc agg atc ttc atg aag gcc tt-3’; rat Forward primer: 5’- atg tca GAA TTC GCC ACC atg gat cca ctg aac ctg tcc tgg tac gat-3’, Reverse primer: 5’- atg tct GCG GCC GCt cag cag tgc aag atc ttc atg aag gcc tt-3’) designed specifically based on the mouse and rat DRD2 cDNA coding regions (Genbank accession Nos: mouse DRD2, NM_010077.3; rat DRD2, NM_012547.1). The mouse and rat brain cDNAs (Clontech) were used as the templates for cDNA amplifications.

### Calcium Mobilization Assay

HEK293 cells transiently transfected with either G_qi5_, DRD2 (human, mouse, and rat), GPR139 (human, mouse, and rat) together or alone, using Fugene HD reagent (Promega, Madison, WI, United States). For 10 cm dish, total of 10 μg of DNA were used for each transfection. For co-expression of GPR139 and DRD2, 5 μg each of the DNAs were used. For co-transfection of DRD2 and G_qi5_, 7.5 μg of DRD2, and 2.5 μg of G_qi5_ were used. The transfected cells were grown in DMEM F-12K culture media (Corning, Corning, NY, United States) containing 10% fetal bovine serum (FBS), 1× penicillin/streptomycin, 1× sodium pyruvate, 20 mM HEPES. Twenty-four hours after the transfection, cells were detached with 0.25% trypsin/2.25 mM EDTA and resuspended in plating media DMEM high glucose (Hyclone) containing 10% FBS, 1× penicillin/streptomycin, 1× sodium pyruvate, 20 mM HEPES. Cells were seeded at 50 μl/well (50,000 cells/well) in poly-D-lysine-coated, black-walled, clear-bottom 96-well tissue culture plates and incubated overnight at 37°C, 5% CO2. Forty-eight hours after the transfection, cell culture medium was aspirated and cells were loaded with 1× BD calcium loading dye (Becton Dickinson, Franklin Lakes, NJ, United States) solution at 100 μl/well and incubated at 37°C, 5% CO2 for 45 min. Compounds L-741,626, JNJ-3792165, and JNJ-63533054 dilutions were prepared in HBSS from 10 mM dimethyl sulfoxide (DMSO) stocks, while Quinpirole dilutions were prepared from 0.1 mM HCl stocks. Cells were treated with 10 μM of DRD2 antagonist L-741,626 or GPR139 antagonist JNJ-2165 for 10 min. Log dilution of 10 μM of DRD2 agonist Quinpirole and GPR139 agonist JNJ-3054 (20 μl) were done on the Fluorometric Imaging Plate Reader Tetra (FLIPR, Molecular Devices, Sunnyvale, CA, United States) and changes in fluorescence that reflect calcium mobilization were monitored at 1-s intervals for 90 s, followed by 3-s intervals for 60 s (excitation wavelength = 470–495 nm, emission wavelength = 515–575 nm). Data were exported as the difference between maximum and minimum fluorescence observed for each well. Data were expressed as relative fluorescence units (RFU). Results were calculated using non-linear regression to determine agonist EC_50_ values (expressed as mean ± S.E.M., Graphpad Prism 7 software, San Diego, CA, United States).

## Results

### GPR139 and DRD2 Share Similarities in Gene Expression Distribution Patterns

**FIGURE 1:**
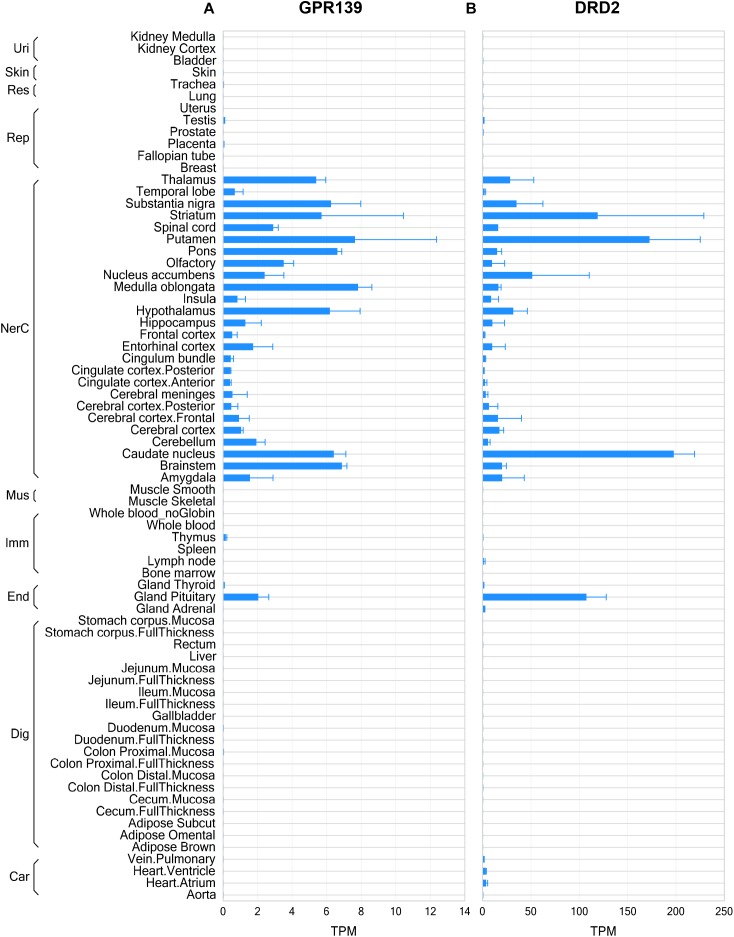
Gene expression of GPR139 and dopamine D2 receptor (DRD2) in human tissues by RNA sequencing. RNA sequencing data showing GPR139 **(A)** and DRD2 **(B)** mRNA expression in human tissues. Expression level is presented as transcripts per kilobase million (TPM). Tissues are grouped by organ systems: Uri, urinary; Res, respiratory; Rep, reproductive; NerC, central nervous; Mus. Muscular; Imm, lymphatic; End, endocrine; Dig, digestive; Car, cardiovascular.

The RNA sequencing data from human tissues revealed that GPR139 and DRD2 exhibited some similarities in their distribution patterns in the CNS, such as olfactory, basal ganglia (including caudate nucleus, putamen, and nucleus accumbens), thalamus, hypothalamus, substantia nigra, medulla, and pons as well as pituitary gland (Figure 1A,B). Data from mouse and rat tissues are consistent with that of human, showing similar distribution patterns in CNS regions and in pituitary gland (Supplementary Figures S1, S2). It is noteworthy that DRD2 expression levels are much higher than GPR139 across all tissues and in all species. Besides, GPR139 expression was more abundant in rat pituitary compared to human and mouse pituitary tissues.

### GPR139 and DRD2 Co-express in the Same Cells of the Brain

**FIGURE 2:**
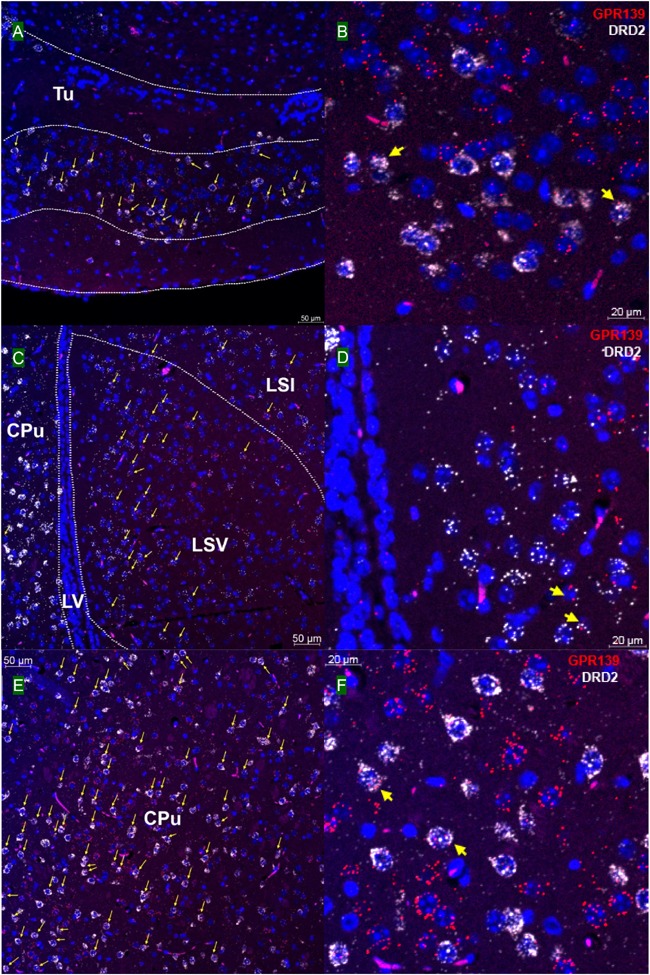
Double *in situ* hybridization of GPR139 (red) and DRD2 (white) mRNA in mouse telencephalon. Yellow arrows indicate colocalization of GPR139 and DRD2 mRNA in the same cells, including olfactory tubercle (Tu, **A**), ventral part of lateral septal nucleus (LSV, **C**), and lateral region of caudate putamen (CPu, **E**). Magnified images of Tu **(B)**, LSV**(D)**, and CPu **(F)** are shown with yellow arrows indicating example of colocalization signals. The nuclei of cells were stained with DAPI (blue). LSI, lateral septal nucleus, intermediate part; LV, lateral ventricle.

**FIGURE 3:**
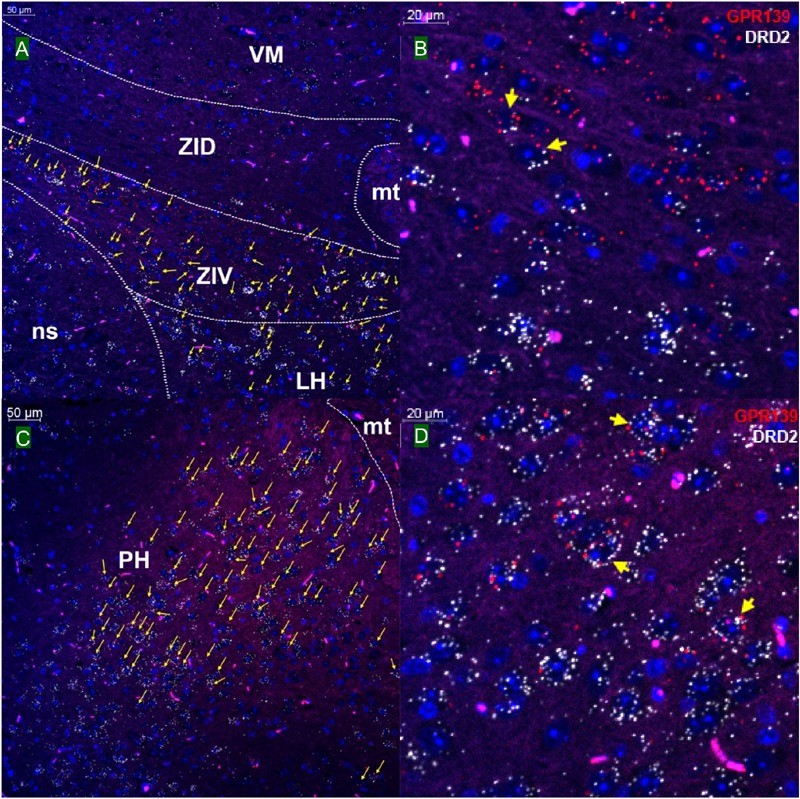
Double *in situ* hybridization of GPR139 (red) and DRD2 (white) mRNA in mouse diencephalon. Yellow arrows indicate colocalization of GPR139 and DRD2 mRNA in the same cells, including ventral part of zona incerta (ZIV, **A**) and posterior hypothalamus (PH, **C**). Magnified images of ZIV **(B)**, PH**(D)** are shown with yellow arrows indicating example of colocalization signals. The nuclei of cells were stained with DAPI (blue). VM, ventromedial thalamic area; ZID, zona incerta, dorsal part; mt, mammillothalamic tract; ns, nigrostriatal bundle; LH, lateral hypothalamic area.

**FIGURE 4:**
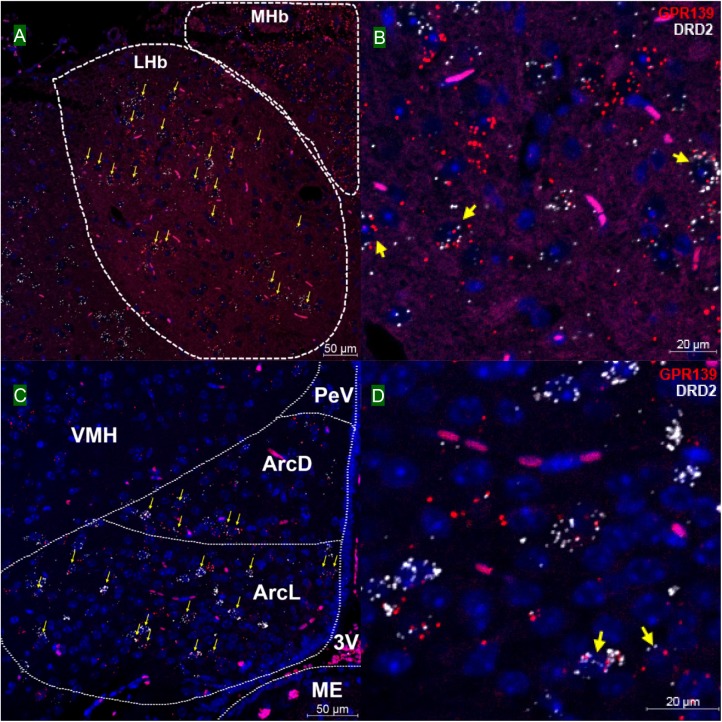
Double *in situ* hybridization of GPR139 (red) and DRD2 (white) mRNA in mouse diencephalon (continued). Yellow arrows indicate colocalization of GPR139 and DRD2 mRNA in the same cells, including lateral habenular nucleus (LHb, **A**) and both dorsal and lateral part of arcuate hypothalamic nucleus (ArcD and ArcL, **C**). Magnified images of LHb **(B)**, ArcL**(D)** are shown with yellow arrows indicating example of colocalization signals. The nuclei of cells were stained with DAPI (blue). MHb, medial habenular nucleus; VMH, ventromedial hypothalamic nucleus; PeV, periventricular hypothalamic nucleus; 3V, 3rd ventricle; ME, median eminence.

**FIGURE 5:**
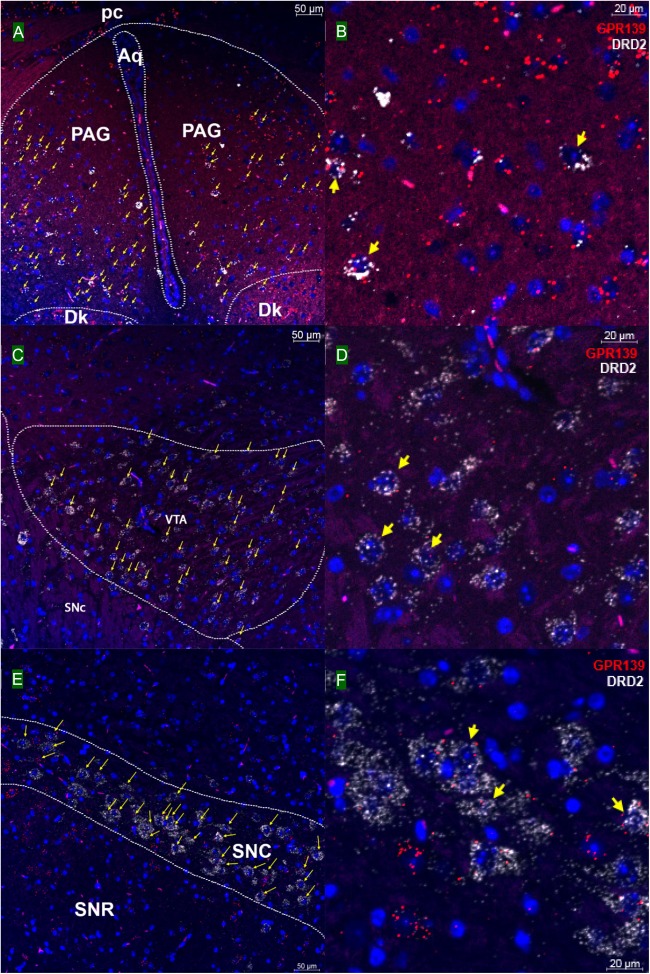
Double *in situ* hybridization of GPR139 (red) and DRD2 (white) mRNA in mouse mid brain. Yellow arrows indicate colocalization of GPR139 and DRD2 mRNA in the same cells, including periaqueductal gray (PAG, **A**), ventral tegmental area (VTA, **C**), and compact part of substantia nigra (SNC, **E**). Magnified images of PAG **(B)**, VTA**(D)**, and SNC **(F)** are shown with yellow arrows indicating example of colocalization signals. The nuclei of cells were stained with DAPI (blue). pc, posterior commissure; Aq, aqueduct; Dk, nucleus of Darkschewitsch; SNR, substantia nigra, reticular part.

**FIGURE 6:**
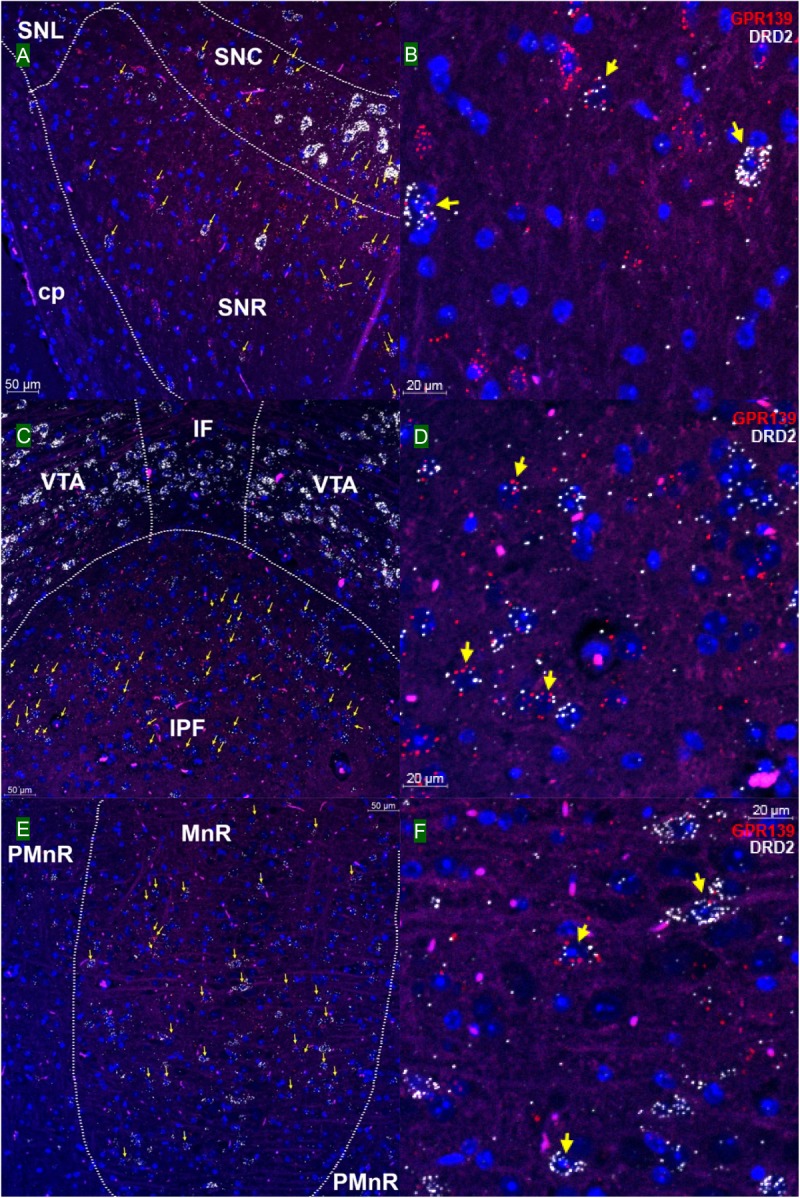
Double *in situ* hybridization of GPR139 (red) and DRD2 (white) mRNA in mouse mid brain (continued). Yellow arrows indicate colocalization of GPR139 and DRD2 mRNA in the same cells, including reticular part of substantia nigra (SNR, **A**), interpeduncular fossa (IPF, **C**) and median raphe nucleus (MnR, **E**). Magnified images of SNR **(B)**, IPF**(D)**, and MnR**(F)** are shown with yellow arrows indicating example of colocalization signals. The nuclei of cells were stained with DAPI (blue). SNC, substantia nigra, compact part; SNL, substantia nigra, lateral part; cp, cerebral, peduncle, basal part; VTA, ventral tegmental area; IF, interfascicular nucleus; PMnR, paramedian raphe nucleus.

We then investigated whether GPR139 and DRD2 co-express in the same cells of mouse brain. Double fluorescent *in situ* hybridization revealed cellular co-expression of these two receptors’ mRNA throughout the brain. In the telencephalon, moderate level of colocalization of GPR139 and DRD2 mRNA were observed in several regions, including olfactory tubercle (Tu, Figure 2A,B), ventral part of lateral septal nucleus (LSV, Figure 2C,D), and lateral region of caudate putamen (CPu, Figure 2E,F). In the diencephalon, GPR139 and DRD2 mRNA co-express in the same cells of ventral part of zona incerta (ZIV, Figure 3A,B), and posterior hypothalamus (PH, Figure 3C,D). While low level of colocalization were observed in lateral habenular nucleus (LHb, Figure 4A,B) and both dorsal and lateral part of arcuate hypothalamic nucleus (ArcD and ArcL, Figure 4C,D). In the midbrain, moderate level of co-expression of GPR139 and DRD2 mRNA in the same cells were found in periaqueductal gray (PAG, Figure 5A,B), ventral tegmental area (VTA, Figure 5C,D), and compact part of substantia nigra (SNC, Figure 5E,F). Additionally, low levels of co-expression in the same cells were also found in the reticular part of substantia nigra (SNR, Figure 6A,B), interpeduncular fossa (IPF, Figure 6C,D) and median raphe nucleus (MnR, Figure 6E,F).

Our *in situ* hybridization results confirmed the previous report that high expression of GPR139 mRNA was observed in medial habenula (MHb, Figure 4A) and lateral septum (LSV, Figure 2C,D), where GPR139 immunoreactivity are also detected (Liu et al., 2015). High expression of GPR139 mRNA has also been confirmed in caudate putamen (Figure 2E,F), zona incerta (ZIV, Figure 3A,B), and median part of medial mammalian nucleus (data not shown). Moderate expression was observed in the lateral habenula (LHb, Figure 4A,B), parafascicular thalamic nucleus (data not shown), arcuate hypothalamus (Figure 4C,D), the nucleus of Darkschewitsch and the medial vestibular nucleus (data not shown) (Matsuo et al., 2005). We also found that GPR139 mRNA was moderately expressed in the granular layer of the dentate gyrus in hippocampus (GrDG, Supplementary Figure S3A) and paraventricular hypothalamic nucleus (PVN, Supplementary Figure S3C). High level of GPR139 mRNA expression was also detected in pyramidal cell layer of the hippocampus (Py, Supplementary Figure S3B). No co-expression of GPR139 and DRD2 were detected among these regions. The distribution pattern of DRD2 mRNA reported in the present study are also consistent with literatures (Mansour et al., 1990; Gehlert et al., 1992; Missale et al., 1998; Beaulieu and Gainetdinov, 2011). As negative controls, negative signals of GPR139 and DRD2 mRNA in representative brain regions are shown in Supplementary Figure S6.

### GPR139 and DRD2 Co-express in the Same Cells of Pituitary

**FIGURE 7:**
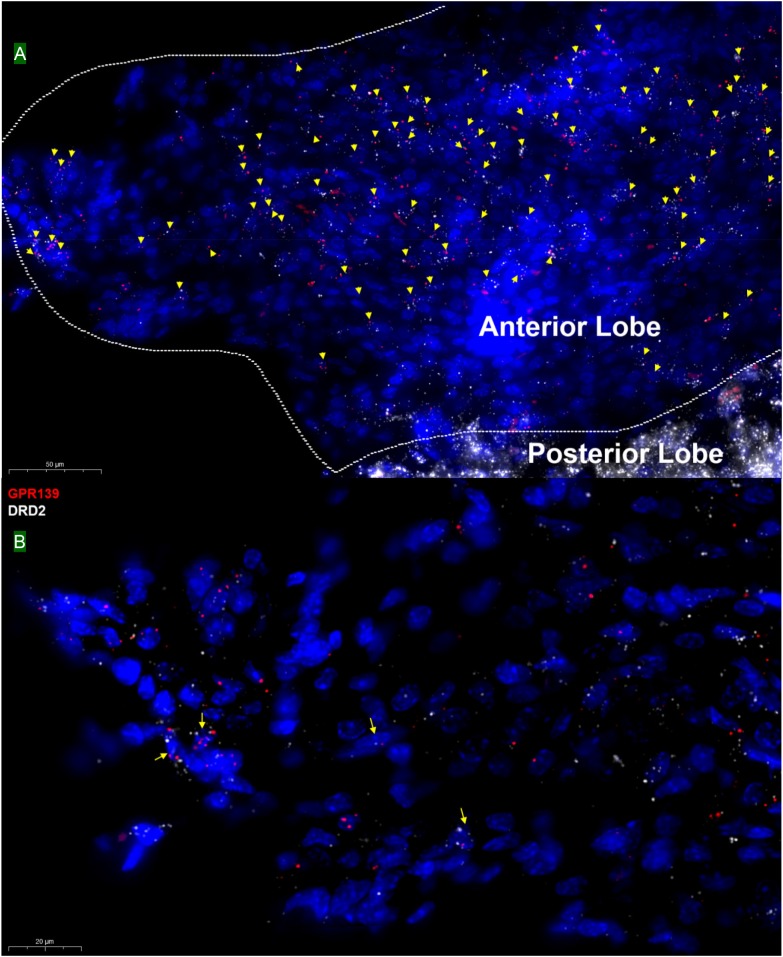
Double *in situ* hybridization of GPR139 (red) and DRD2 (white) mRNA in mouse pituitary. Yellow arrows indicate colocalization of GPR139 and DRD2 mRNA in the same cells of the anterior lobe **(A)**. Magnified image **(B)** is shown with yellow arrow indicating example of colocalization signals. The nuclei of cells were stained with DAPI (blue).

Double *in situ* hybridization was also applied on mouse pituitary sections. GPR139 mRNA was observed exclusively in the anterior lobe of pituitary with moderate co-expression with DRD2 mRNA in the same cells throughout the entire anterior lobe of pituitary (Figure 7A,B; Mansour et al., 1990).

### DRD2 Activates Calcium Mobilization Through GPR139 *in vitro*

**FIGURE 8:**
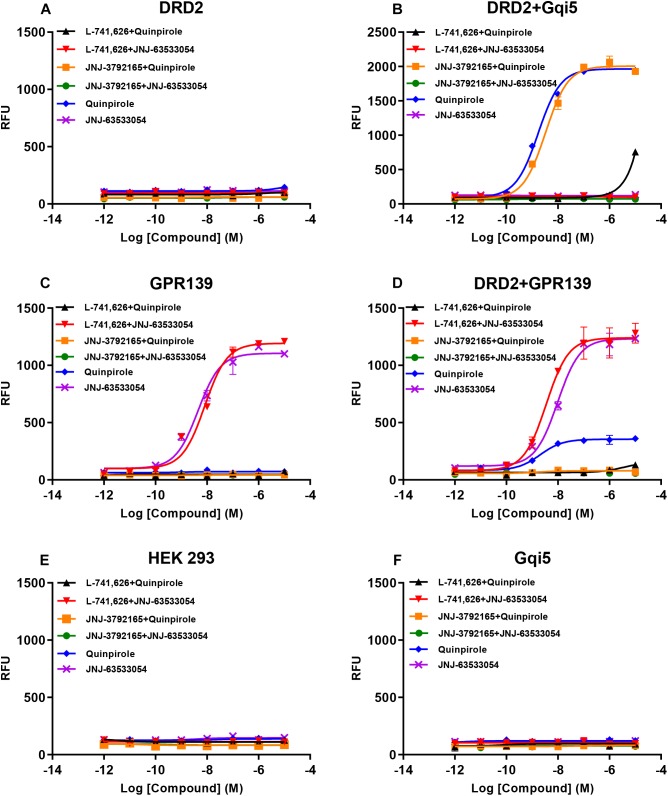
Concentration responses of the DRD2 agonist quinpirole and GPR139 agonist JNJ-63533054 calcium mobilization with or without 10 μM of the DRD2 antagonist L-741,626 or GPR139 antagonist JNJ-3792165 in HEK 293 cells transfected with only human DRD2 **(A)**, human DRD2 and G_qi5_ **(B)**, only human GPR139 **(C)**, human DRD2 and GPR139 **(D)**, no receptors **(E)**, or only Gqi5 **(F)**. Only GPR139 **(C)** but not DRD2 **(A)** transfected alone elicited Ca^2+^ mobilization. DRD2 was able to elicit Ca^2+^ mobilization when co-transfected with either G_qi5_ **(B)** or GPR139 **(D)**. Neither control HEK293 cells **(E)** nor HEK293 cells transfected only with G_qi5_ **(F)** elicited Ca^2+^ mobilization. Error bars represent standard error of the mean of duplicate measurements for each point (*n* = 2).

Based on the relatively high level of cellular colocalization between GPR139 and DRD2 mRNAs, we studied their *in vitro* functional interaction in HEK293 cells transiently transfected with human GPR139 and DRD2. Consistent with the G_i_ transduction pathway of DRD2, the DRD2 agonist quinpirole was not able to elicit a calcium response in HEK293 cells transfected with DRD2 alone (blue line, Figure 8A). Gqi5 is a chimeric G-protein of Gq protein with its last 5 amino acid residues at the very C-terminus replaced by the last C-terminal 5 residues of G_i_ protein (Conklin et al., 1993). When co-expressed with a GPCRs normally signal through G_i_ signaling pathway, G_qi5_ is able to convert the G_i_ signal of GPCRs into G_q_ signal pathway, namely cause calcium mobilization. When co-transfected with G_qi5_, quinpirole was able to stimulate a calcium response in DRD2 expressing cells in the low nM range (EC_50_ = 1.72 ± 1.11 nM, blue line, Figure 8B). This effect on calcium mobilization was blocked by the DRD2 antagonist L-741,626 (black line, Figure 8B). GPR139 is coupled to G_q_ coupled receptor and therefore, the GPR139 agonist, JNJ-63533054, was able to elicit calcium mobilization in HEK293 cells transfected with GPR139 alone, as previously reported (EC_50_ = 4.46 ± 1.26 nM, purple line, Figure 8C). This effect was antagonized by the GPR139 antagonist JNJ-3792165 (green line, Figure 8C). When DRD2 and GPR139 were co-transfected, the DRD2 agonist quinpirole stimulated calcium mobilization in the absence of G_qi5_ (EC_50_ = 2.04 ± 1.32 nM, blue line, Figure 8D). This response was antagonized by either the DRD2 antagonist L-741,626 (black line, Figure 8D) or the GPR139 antagonist JNJ-3792165 (yellow line, Figure 8D). No calcium response was observed by either agonist in un-transfected HEK 293 cells or cells only expressing G_qi5_ (Figure 8E,F). Similar interaction between GPR139 and DRD2 were observed using rat or mouse orthologue from each receptor (Supplementary Figures S4, S5). These results demonstrated an *in vitro* functional interaction between dopamine DRD2 and GPR139 in HEK293 cells.

## Discussion

Using the highly sensitive and selective RNAscope^®^ technique, we demonstrated co-expression in the same cells of GPR139 and DRD2 mRNA in the ventral tegmental area (VTA, Figure 5C,D), olfactory tubercle (Tu, Figure 2A,B), substantia nigra pars compacta (SNC, Figure 5E,F), caudate and putamen (CPu, Figure 2E,F), arcuate hypothalamic nucleus (ArcH, Figure 4C,D) as well as the anterior lobe of pituitary gland (Figure 7A,B). All these brain regions are part of the dopaminergic afferent and efferent signaling network. Interestingly, in light of the DRD2/GPR139 mRNAs co-expression found in the mesolimbic pathway (projects from VTA to nucleus accumbens and Tu) (Ikemoto, 2010), a recent preclinical study using the selective GPR139 agonist JNJ-63533054 showed that GPR139 receptor activation reverses key addiction-like behaviors in dependent animals (Kononoff et al., 2018). Another GPR139 agonist 4-oxo-3,4-dihydro-1,2,3-benzotriazine has been reported to improve social withdrawal in a preclinical model of the negative symptom of schizophrenia (Atienza et al., 2018), involving the mesocortical pathway (initiate from VTA but instead project to the prefrontal cortex) (Ekhtiari and Paulus, 2016). A recent study reported that the GPR139 agonist TC-O 9311 (structurally identical to compound 1a used in this study) protected primary mesencephalic dopamine neurons against 1-methyl-4-phenylpyridinium (MPP+)-mediated degeneration, indicating a potential role of GPR139 in neuroprotection and Parkinson’s disease (Bayer Andersen et al., 2016). Meanwhile, it has also been reported that the activation of DRD2 by its agonist quinpirole protected dopamine neurons from MPP+ induced cell death (Wiemerslage et al., 2013). The co-expression of GPR139 and DRD2 mRNA in the same cells of nigrostriatal pathway (projects from SNC to CPu) and their *in vitro* signaling interaction may indicate the possibility of their functional interaction in terms of neuroprotection. And lastly the co-expression of GPR139 and DRD2 mRNA in the same cells of ArcH and the anterior lobe of pituitary gland match the tuberoinfundibular pathway (projects from ArcH to the median eminence which controls the secretion of prolactin from the anterior lobe of pituitary gland). While the physiological role of GPR139 in prolactin regulation has not been reported, dopamine is known as prolactin inhibitor and reduces prolactin release by binding to DRD2 expressed on the cell membrane of the lactotroph in the pituitary (Ben-Jonathan and Hnasko, 2001). Taken together, the co-expression of GPR139 and DRD2 mRNA in the same cells within dopaminergic signaling pathways may indicate a potential functional interaction between these two receptors under physiological conditions, which merits further *in vivo* investigation.

Co-expression of GPR139 and DRD2 mRNA in the same cells has also been demonstrated in lateral habenula (LHb, Figure 4A,B), lateral septum (LSV, Figure 2C,D), interpeduncular fossa (IPF, Figure 6C,D), and median raphe nucleus (MnR, Figure 6E,F). Specifically, the functions of lateral habenula has been attributed to several common mechanisms (Hikosaka, 2010). It is proposed that the LHb responds to the negative stimulus (e.g., failure and punishment) that derives from basal ganglia and transmit the signaling to dopaminergic neurons located in SNC and VTA, which ultimately leads to motor suppression (aversive outcome). On the other hand, painful or stressful stimulus activates LHb through limbic system (e.g., septum and medial frontal cortex), and LHb further inhibits both dopaminergic neurons in SNC and VTA, as well as serotonergic neurons in raphe nuclei (dorsal and medial, DnR, and MnR) through the projection from interpeduncular nucleus (IPN). These results may also suggest future research directions to their potential interaction in terms of habenula based neurotransmitter regulation and physiological functions.

Apart from the previously reported regions, our current study also revealed several other brain areas with GPR139 mRNA expression, such as dentate gyrus in hippocampus (GrDG, Supplementary Figure S3A), pyramidal cell layer of the hippocampus (Py, Supplementary Figure S3B) and paraventricular hypothalamic nucleus (PVN, Supplementary Figure S3C), which has not been reported previously. We also demonstrated the site of GPR139 mRNA expression in the pituitary (Figure 7A,B). The expression of GPR139 mRNA in the PVN and anterior lobe of pituitary may suggest a possible role for GPR139 in the hypothalamic-pituitary-adrenal (HPA) axis (Malenka et al., 2009), however this will remain to be determined.

Nonetheless, our previous immunohistochemistry results only showed positive GPR139 protein expression in medial habenula and lateral septum (Liu et al., 2015), which may indicate the variation of sensitivity between the methods detecting mRNA and protein expressions. In the current study, the RNAscope^®^ technique utilizes two independent probes, which hybridize to their own target sequence in tandem for signal amplification. The situation that two independent probes hybridize to a non-specific target right next to each other is highly unlikely, which ensures selective amplification of target-specific signals and elimination of background noise. Moreover, GPR139 is activated by the essential amino acids L-Trp and L-Phe with EC_50_ values in the 30- to 300-μM range, which is consistent with the physiologic concentrations of both amino acids (Liu et al., 2015). Thus, it is probable that GPR139 is constitutively activated under native *in vivo* conditions. The constant activation of GPR139 may result in constant desensitization and receptor internalization, which may further disturb antibody binding and protein detection in regions with weak to moderate mRNA expression levels. Additional studies are required to prove the protein expression of GPR139 in those regions.

Once we observed that GPR139 and DRD2 appear to be co-expressed in the same cells in various brain regions, we investigated a possible *in vitro* interaction between these two receptors. We observed an intriguing signaling interaction where the presence of GPR139 was required to observe DRD2 agonist-elicited calcium mobilization when co-transfected with GPR139 in HEK 293 cells. This effect was blocked by either a DRD2 antagonist or a GPR139 antagonist (Figure 8D), indicating that the DRD2 agonist-stimulated Ca^2+^ signaling involves the activation of both DRD2 and GPR139. Just recently our group reported another *in vitro* signaling interaction between GPR139 and melanocortin receptors (MC_3_R, MC_4_R, and MC_5_R) (Nepomuceno et al., 2018). One of the possible mechanisms is the formation of heteroreceptor complexes between these receptors resulting in calcium signaling cross talk (Werry et al., 2003). The allosteric communication and functional cross talk between GPCR homo- and heterodimerization have been extensively reported (Borroto-Escuela et al., 2014, 2017). Specifically, DRD2 forms heteroreceptor complexes with other GPCRs or ion channel receptors, such as A_2A_R (adenosine 2A receptor)-DRD2 (Trifilieff et al., 2011), A_2A_R-DRD2-mGluR5 (metabotropic glutamate receptor 5) (Cabello et al., 2009), A2AR-DRD2-Sigma1R (Pinton et al., 2015), DRD2-NMDAR (N-methyl-D-aspartate receptor) (Liu et al., 2006), DRD2-5-HT_2A_R (serotonin 2A receptor) (Albizu et al., 2011), and DRD2-OXTR (oxytocin receptor) (de la Mora et al., 2016). These DRD2 heteroreceptor complexes are mainly found in the striatum (Borroto-Escuela et al., 2017). Additional studies using biochemical approach, such as fluorescence resonance energy transfer technique, or co-immunoprecipitation using specific antibodies for GPR139 and DRD2 would further support the interaction between these two receptors.

In conclusion, we found that GPR139 and the dopamine DRD2 mRNA co-express in the same cells of certain brain regions and that they functionally interact in an *in vitro* system. It is worthwhile to mention that there was one phase 1 clinical trial (NCT02959892) studied the effect of a GPR139 agonist (TAK-041) on amphetamine induced dopamine release with no result posted. It remains to be determined if the same intriguing *in vitro* signaling interaction will be observed *in vivo*.

## Author Contributions

LW, PB, TL, and CL participated in research design. LW, GL, CK, and XY conducted the experiments. LW, GL, and XY performed the data analysis. LW, GL, XY, AH, PB, TL, and CL wrote or contributed to the writing of the manuscript.

## Conflict of Interest Statement

All authors are paid employees at Janssen Research and Development, LLC.
