# Supplementary material for: GPR139 and Dopamine D2 Receptor Co-express in the Same Cells of the Brain and May Functionally Interact
Source: Front Neurosci. 2019 Mar 26;13:281. doi: 10.3389/fnins.2019.00281 (PMC6443882; doi:10.3389/fnins.2019.00281)
Supplement: Supplementary file 1 [file Data_Sheet_1.docx]

**Title: GPR139 and dopamine D2 receptor co-localize in the brain and may functionally interact**

**Supplemental Data**

**Fig. S1**

**A**

**GPR139**

**DRD2**

**B**

**Figure S1. Gene expression of GPR139 and dopamine D2 receptor (DRD2) in mouse tissues by RNA sequencing.** RNA sequencing data showing GPR139 (**A**) and DRD2 (**B**) mRNA expression in mouse tissues. Expression level is presented as transcripts per kilobase million (TPM). Tissues are grouped by organ systems: Uri, urinary; Res, respiratory; Rep, reproductive; NerC, central nervous; Mus. Muscular; Imm, lymphatic; End, endocrine; Dig, digestive; Car, cardiovascular.

**Fig. S2**

**DRD2**

**B**

**A**

**GPR139**

**Figure S2. Gene expression of GPR139 and DRD2 in rat tissues by RNA sequencing.** RNA sequencing data showing GPR139 (**A**) and DRD2 (**B**) mRNA expression in rat tissues. Expression level is presented as transcripts per kilobase million (TPM). Tissues are grouped by organ systems: Uri, urinary; Res, respiratory; Rep, reproductive; NerC, central nervous; Mus. Muscular; Imm, lymphatic; End, endocrine; Dig, digestive; Car, cardiovascular.

**Fig. S3**


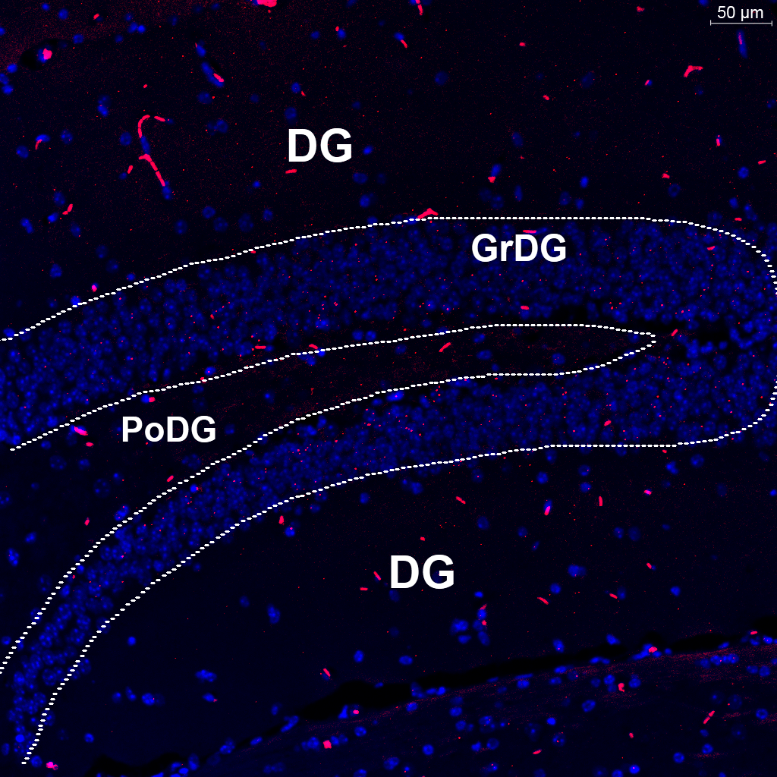


A

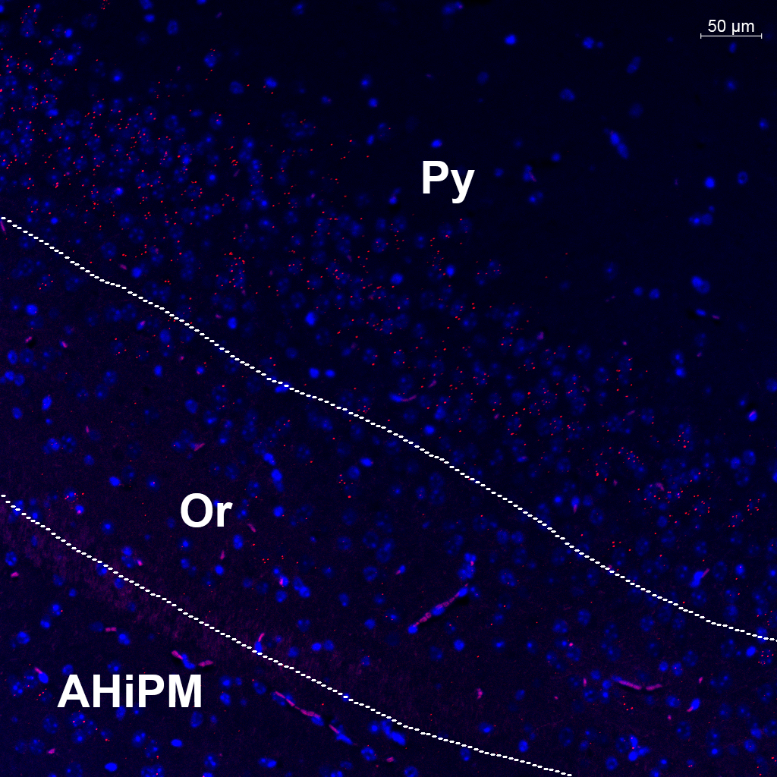


B

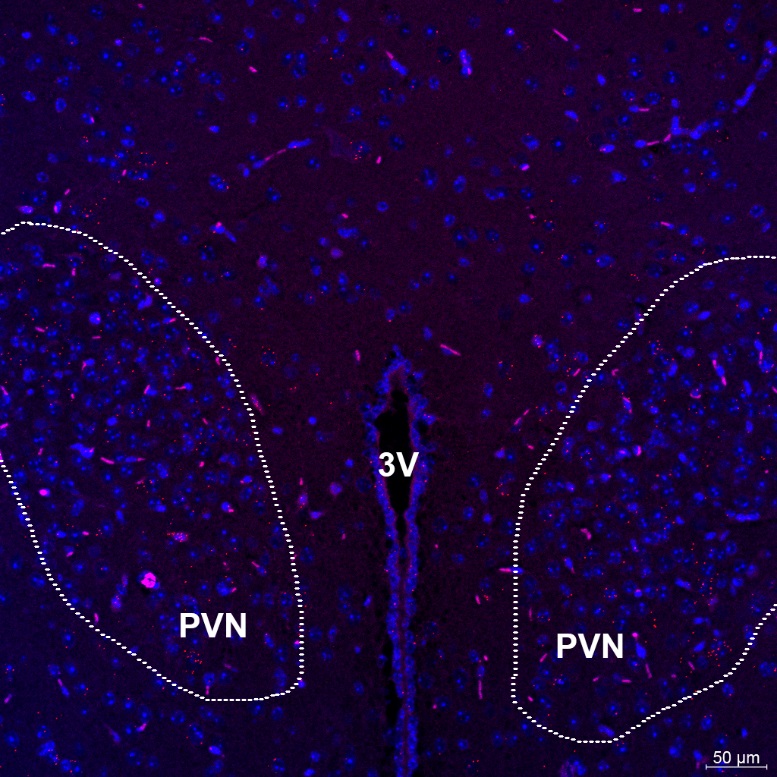


C

**Figure S3. *In situ* hybridization of GPR139 (cy3, red) mRNA in mouse brain**. Expression of GPR139 mRNA is found in granular layer of the dentate gyrus in hippocampus (GrDG, **A**), pyramidal cell layer of the hippocampus (Py, **B**) and paraventricular hypothalamic nucleus (PVN, **Fig. C**). The nuclei of cells were stained with DAPI (blue). DG, dentate gyrus; PoDG, polymorph layer of the dentate grus; Or, oriens layer of the hippocampus; AHiPM, amygdalohippocampal area, posteromedial part; 3V, 3^rd^ ventricle.

**Fig. S4**

**Figure S4. Concentration responses of the DRD2 agonist quinpirole and GPR139 agonist JNJ-63533054 calcium mobilization with or without 10 μM of the DRD2 antagonist L-741,626 or GPR139 antagonist JNJ-3792165 in HEK 293 cells transfected with only mouse DRD2 (A), mouse DRD2 and G_qi5_ (B), only mouse GPR139 (C), mouse DRD2 and GPR139 (D).** DRD2 was able to elicit Ca^2+^ mobilization when co-transfected with either Gqi5 or GPR139. Error bars represent standard error of the mean of duplicate measurements for each point. (n = 2)

**Fig. S5**

**Figure S5. Concentration responses of the DRD2 agonist quinpirole and GPR139 agonist JNJ-63533054 calcium mobilization with or without 10 μM of the DRD2 antagonist L-741,626 or GPR139 antagonist JNJ-3792165 in HEK 293 cells transfected with only rat DRD2 (A), rat DRD2 and G_qi5_ (B), only rat GPR139 (C), rat DRD2 and GPR139 (D).** DRD2 was able to elicit Ca^2+^ mobilization when co-transfected with either Gqi5 or GPR139. Error bars represent standard error of the mean of duplicate measurements for each point. (n = 2)

**
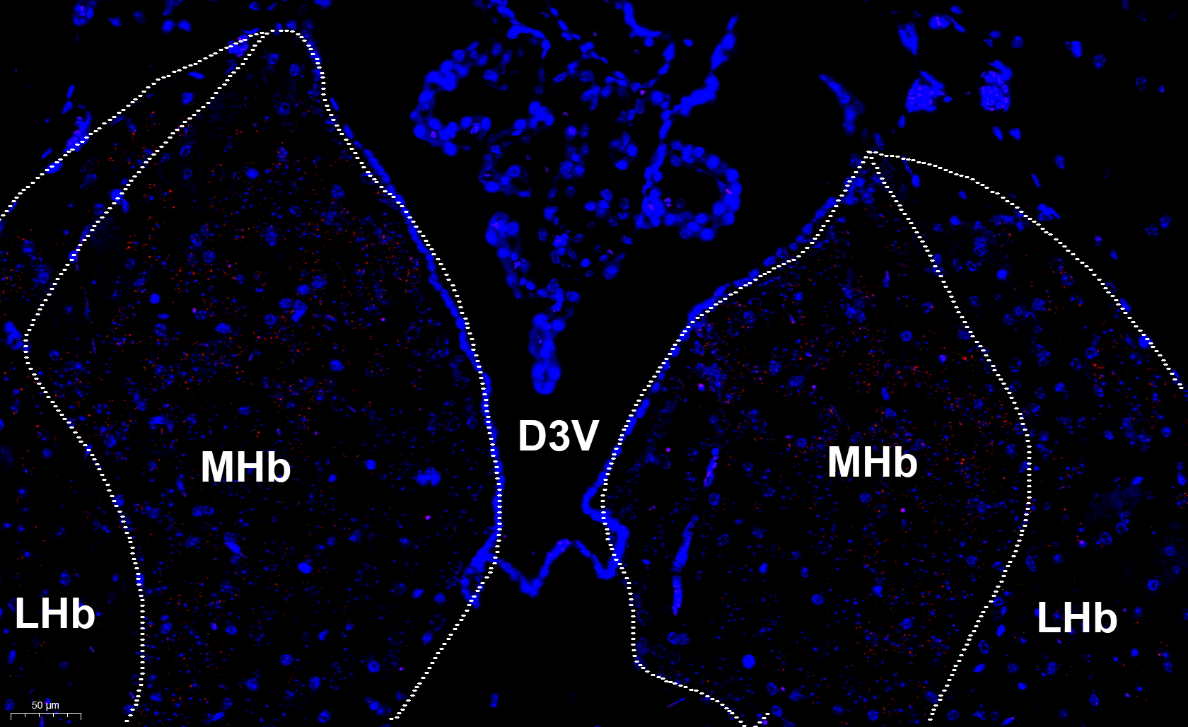
Fig. S6**

A

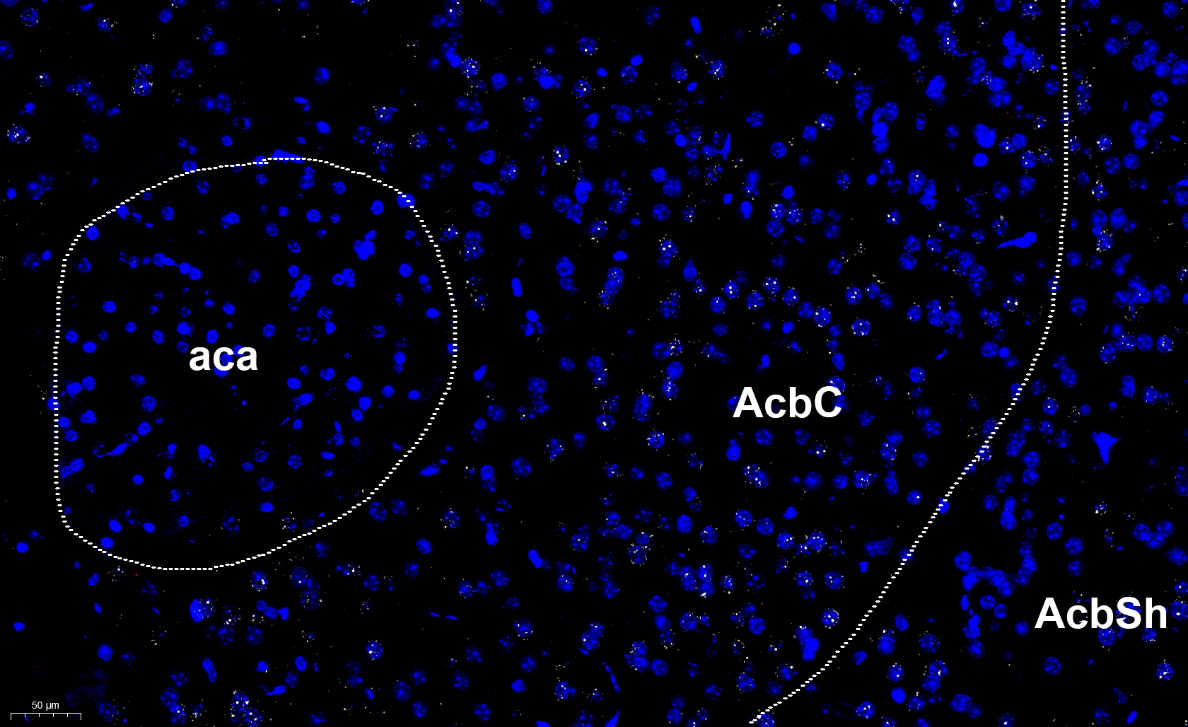


B

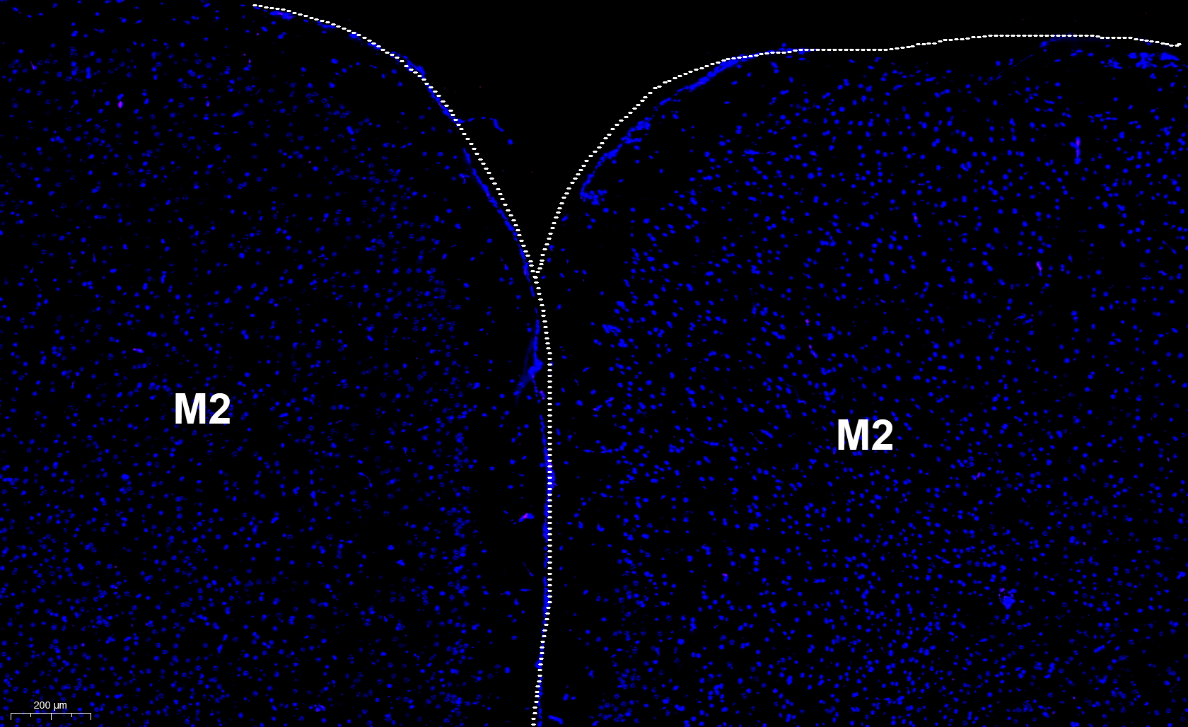


C

**Figure S6. Negative *in situ* hybridization signals of GPR139 (cy3, red) and DRD2 (cy5, white) mRNA in representative mouse brain regions**. Images were acquired using scanned and digitalized brain slides by Pannoramic SCAN II scanner (3DHISTECH Ltd, Budapest, Hungary). Images were processed and analyzed with CaseViewer 2.1 software (3DHISTECH Ltd, Budapest, Hungary) and Zen Lit 2.3 software (Blue Edition, Carl Zeiss Inc., Oberkocken, Germany). Expression of GPR139 but not DRD2 mRNA signals is found in medial habenula (MHb, **A**). Expression of DRD2 but not GPR139 mRNA signals is found in accumbens nucleus core (AcbC, **B**). Expression of neither GPR139 nor DRD2 mRNA signals is found in secondary motor cortex (M2, **C**). The nuclei of cells were stained with DAPI (blue). D3V, dorsal 3^rd^ ventricle; LHb, lateral habenula; aca, anterior commissure, anterior part; AcbSh, accumbens nucleus, shell.
